# A gene regulatory network inference model based on pseudo-siamese network

**DOI:** 10.1186/s12859-023-05253-9

**Published:** 2023-04-21

**Authors:** Qian Wang, Maozu Guo, Jian Chen, Ran Duan

**Affiliations:** 1 School of Electrical and Information Engineering, Beijing University of Civil Engineering and Architecture, Beijing, China; 2 College of Agronomy and Biotechnology, China Agricultural University, Beijing, China

**Keywords:** Gene regulatory network, Pseudo-siamese network, Deep learning, Time-series expression, Maize

## Abstract

**Motivation:**

Gene regulatory networks (GRNs) arise from the intricate interactions between transcription factors (TFs) and their target genes during the growth and development of organisms. The inference of GRNs can unveil the underlying gene interactions in living systems and facilitate the investigation of the relationship between gene expression patterns and phenotypic traits. Although several machine-learning models have been proposed for inferring GRNs from single-cell RNA sequencing (scRNA-seq) data, some of these models, such as Boolean and tree-based networks, suffer from sensitivity to noise and may encounter difficulties in handling the high noise and dimensionality of actual scRNA-seq data, as well as the sparse nature of gene regulation relationships. Thus, inferring large-scale information from GRNs remains a formidable challenge.

**Results:**

This study proposes a multilevel, multi-structure framework called a pseudo-Siamese GRN (PSGRN) for inferring large-scale GRNs from time-series expression datasets. Based on the pseudo-Siamese network, we applied a gated recurrent unit to capture the time features of each TF and target matrix and learn the spatial features of the matrices after merging by applying the DenseNet framework. Finally, we applied a sigmoid function to evaluate interactions. We constructed two maize sub-datasets, including gene expression levels and GRNs, using existing open-source maize multi-omics data and compared them to other GRN inference methods, including GENIE3, GRNBoost2, nonlinear ordinary differential equations, CNNC, and DGRNS. Our results show that PSGRN outperforms state-of-the-art methods. This study proposed a new framework: a PSGRN that allows GRNs to be inferred from scRNA-seq data, elucidating the temporal and spatial features of TFs and their target genes. The results show the model’s robustness and generalization, laying a theoretical foundation for maize genotype-phenotype associations with implications for breeding work.

**Supplementary Information:**

The online version contains supplementary material available at 10.1186/s12859-023-05253-9.

## Introduction

Maize (Zea mays) is an important worldwide food crop and an important human nutrition, animal feed, and bioenergy source. However, with the reduction in global cultivated land area, maize production has become an essential issue in worldwide food security [1]. Bioinformatics can realize in-depth mining and analysis of complex biological processes and has become an indispensable and efficient tool in crop breeding [2]. Therefore, understanding the growth process of maize and exploring the relationship between the genotype and phenotype of maize seeds with the help of bioinformatics methods will help screen for particular traits in maize breeding to improve maize varieties and yield [3–5].

The inference of gene regulatory networks (GRNs) holds great promise for uncovering complex gene-level information and its connection to phenotypic traits, providing solutions for a range of applications, such as medicine [6], biology [7], and agriculture [8]. With the advent of high-throughput sequencing technologies, such as RNA sequencing (RNA-Seq) [9] and chromatin immunoprecipitation followed by sequencing (ChIP-Seq) [10], GRN inference research has advanced considerably, enabling the study of regulatory relationships between genes at the molecular level [11–14]. However, the verification of these relationships typically requires biological experiments, which are constrained by the limited amount of data generated, extended time frames, and high costs in terms of human and material resources [15]. Recent developments in machine learning and computational biology have paved the way for the use of machine learning models to reverse engineer gene expression metrics and infer GRNs in a rapid and efficient manner [16]. A wide range of computational methods has been proposed for GRN inference, including Bayesian networks [17–20], information theory [21–24], and differential equation models [25, 26].

Many existing methods, such as GENIE3 [27], GRNBoost2 [28], TIGRESS [29], and PoLoBag [30], formulate GRNs as regression-based problems, assuming that all regulatory interactions are functional and that changes in the expression levels of genes result from the regulation of specific transcription factors (TFs). However, biological networks exhibit multiple regulatory mechanisms and interactions that only exist at certain times.

In recent years, several supervised learning approaches have been proposed for inferring GRNs by analyzing known regulatory relationships’ features and integrating prior knowledge to infer unknown regulatory relationships. One of the most successful models in this area is CNNC, proposed by Yuan et al. [31]. CNNC encodes the scRNA-seq data of TFs and their targets into histograms of expression data and employs two Convolutional Neural Network (CNN) layers to explore the interactions between each pair. Building on the success of CNNC, Zhao et al. proposed a hybrid deep-learning framework, named DGRNS [32], which utilizes Pearson Correlation Coefficient (PCC) to encode scRNA-seq data and combines Recurrent Neural Network (RNN) and CNNs to train models that can differentiate pairs of genes with known interactions and infer unknown interactions between other pairs of TF-targets. The accuracy and execution speed of both CNNC and DGRNS have attracted significant attention in the bioinformatics community.

Despite the development of numerous models for inferring GRNs, several challenges persist in the field. These include the extraction of relevant features from gene expression data, the practical engineering of these features, and the accurate assessment of gene interactions. Given the complexity of the data sources, such approaches need to be more generalizable across biological mechanisms to achieve widespread applicability.

To address these issues, we proposed a supervised framework, the pseudo-Siamese network-based GRN inference framework (PSGRN), to infer GRNs. Based on this foundation, this study considers GRN inference as an association classification problem affected by multiple factors. In particular, we expressed gene interaction as the correlation between gene expression series and applied the pseudo-Siamese network to automatically learn the time features contained in the series and the spatial features generated after two features have been concatenated. The critical overviews underlying our approach are the feature matrices constructed from genes and capturing the key features to obtain the underlying changes between genes. Co-located genes with similar topological roles in the co-expression network may interact at some stages. These insights enabled us to discover the standard features of gene interactions and infer whether unknown genes have regulatory relationships. We extensively considered the gene expression features and embedded different feature extractors, including gated recurrent units (GRUs) [33] for time-feature learning and DenseNet [34] for spatial-feature learning. After learning the gene expression feature matrix, we can discover the correlation in the gene expression data and infer whether there is a regulatory relationship between other genes. Finally, we checked the efficiency of each PSGRN part. PSGRN significantly improve inference accuracy, robustness, and generalization ability compared to other methods.

This study’s significance realizes the construction of a large-scale GRN by using deep learning and applying it to maize multi-omic data to lay a theoretical foundation for maize genotype-phenotype association and later breeding work.

**Fig. 1:**
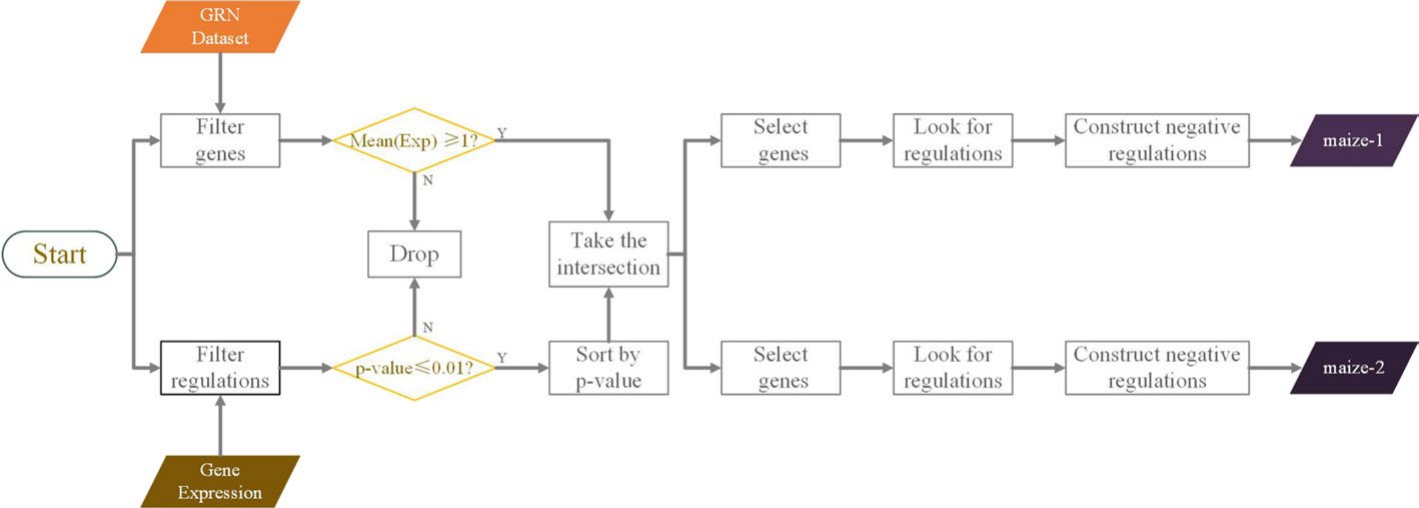
Data processing framework for exploring gene expression in real-world organisms based on the expression data of maize seeds and the regulatory relationships of leaves. Upon preprocessing the gene time expression dataset and the regulatory relationship dataset, we removed the untrusted parts ($mean\left( Exp \right) <1$ and *p*-value$>0.01$) and sorted the regulatory relationships based on their *p*-values. We then selected the genes that appeared in the top 500 and 1000 regulatory relationships and identified the credible regulatory relationships among them. Using this approach, we constructed two sub-datasets, called maize-1 and maize-2, which contain the most reliable regulatory relationships among the expressed genes

## Datasets

We evaluated the performance by applying PSGRN to the gene expression data of maize seeds from the National Center for Biotechnology Information (NCBI) database and comparing it with other GRN inference approaches.

The expression and function of most genes involved in grain development remain unclear [35–37]. Yi et al. [38] conducted RNA-Seq analysis on the nucellus (including the embryo sac) of the maize inbred line B73 and detected 22790 genes, including 1415 TFs. They drew the transcriptome landscape of early maize seed development with high temporal resolution. Covering 22790 gene expressions and 107 time points provides essential resources for future studies on the gene regulation of grain development.

Tu et al. [39] applied ChIP-Seq technology to large-scale transcriptional data technology to analyze TF binding and DNA promoters at the gene level and constructed the GRN in maize leaves based on experimental results; this GRN contains 272627 regulatory relationships, revealing the interaction of 104 TFs with other genes. These results demonstrate the structure, organization principles, and evolution of plant transcriptional regulatory networks, helping to elucidate plant transcriptional regulatory processes.

Previous studies have shown that TFs and their target genes tend to maintain their regulatory relationships across different stages of development in an organism, as observed in eukaryotes like humans [40], mice [41], Escherichia coli [42], and Arabidopsis [43]. Therefore, we applied the known GRN from maize leaves to infer the GRN of maize seeds.

Figure 1 shows the data preprocessing steps. Here, real expression and regulation datasets were obtained from the NCBI database. Co-expression of genes in maize seeds and leaves can be considered part of the GRN of maize seeds. We preprocessed and constructed sub-datasets by eliminating the non-expressed genes and strengthening their regulatory relationships. We constructed two networks at different scales to evaluate the PSGRN generalization.

**Fig. 2:**
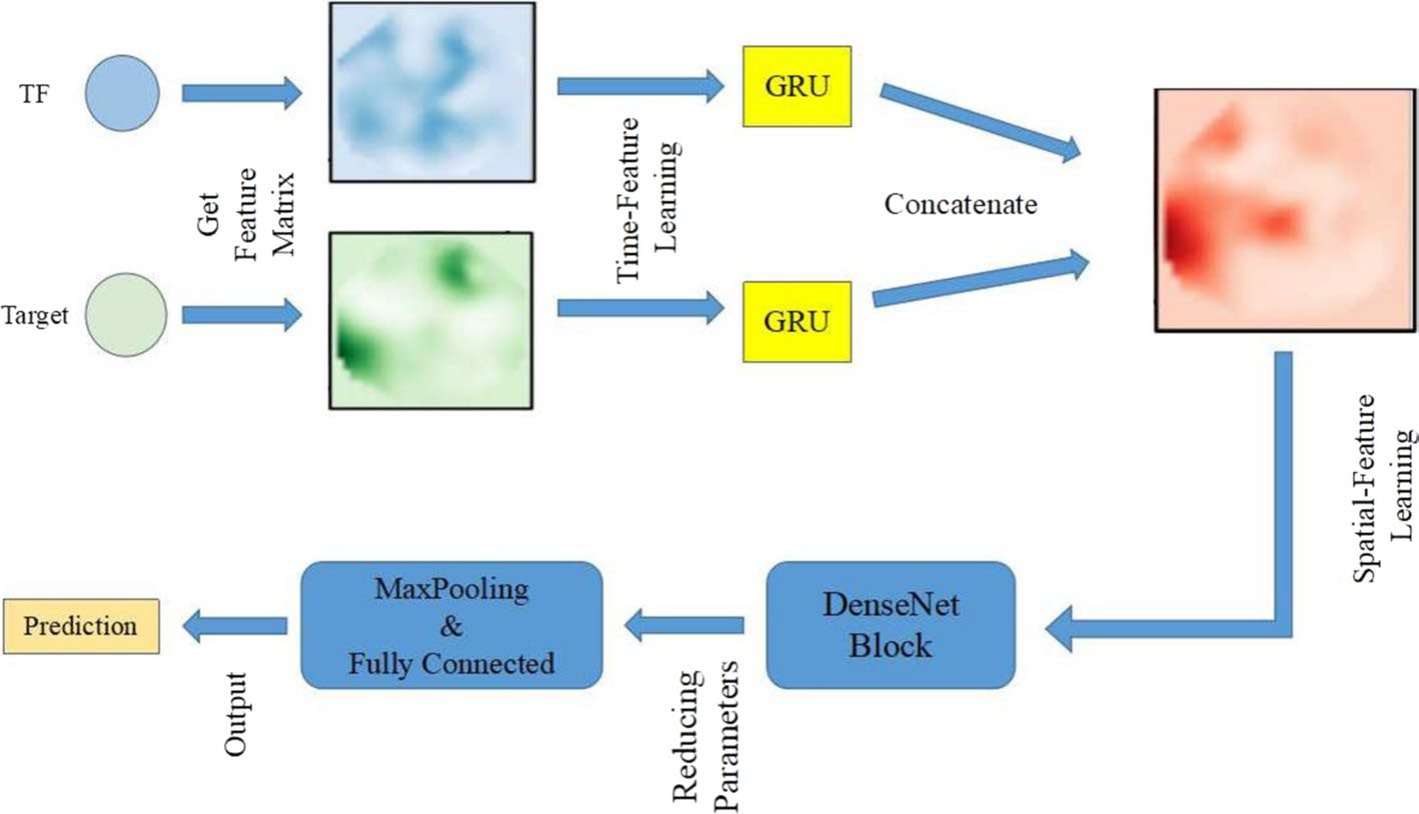
PSGRN framework. PSGRN aim to evaluate the relationships between TFs and target genes using a pseudo-Siamese network consisting of three steps. This model extracts feature matrices from each TF and target gene according to preprocessed datasets. The GRU and DenseNet are applied to obtain hidden time and spatial information. Finally, the regulatory and non-regulatory gene pairs are distinguished, and the prediction results are outputted

This research conducted a comprehensive analysis of two datasets and derived two sub-datasets. The first step involved filtering the gene expression dataset to retain genes with a mean expression level of at least 1.0 ($mean\left( Exp \right) \ge 1.0$) and removing non-expressed genes with a mean expression level below 1.0 ($mean\left( Exp \right) <1.0$). We then applied a *p*-value threshold of 0.01 to identify regulatory relationships among the expressed genes in the GRN dataset. Relationships with a *p*-value of 0.01 or less were considered reliable regulations and were sorted in ascending order of *p*-value. The top 500 and top 1000 genes from this regulation dataset were selected, and the corresponding integrated time series of expressions were designated as maize-1 and maize-2, respectively.

To label regulatory relationships as gold standards, we utilized the data-pair construct process as outlined in the DREAM4 challenge [44]. We listed each potential TF-target relationship pair and assigned a binary label of 1 to pairs with a *p*-value $\le 0.01$ in the GRN dataset. Pairs with *p*-values above this threshold or not shown in the GRN dataset were assigned a label of 0. These labeled regulatory relationships were utilized as gold standards for both maize-1 and maize-2. Table 1 presents a detailed description of the datasets, including the number of genes, the number of regulatory relationships, and the number of TF-target pairs for each dataset.

**Table 1:** Different scales of maize gene datasets introduction

Datasets	Genes$^{\text{a}}$	TFs$^{\text{b}}$	Regulations$^{\text{c}}$	Network density$^{\text{d}}$
Maize-1	506	101	9690	0.190
Maize-2	876	148	16367	0.126

$^{\text{a}}$Number of genes in the datasets (containing TFs)

$^{\text{b}}$Number of TFs contained in gene expression dataset

$^{\text{c}}$Number of regulatory relationships contained in standard network

$^{\text{d}}$Calculate Network Density through Regulations/(TFs*Genes)

## Method

The framework is illustrated in Fig. 2. PSGRN aim to evaluate the relationship between TFs and their target genes using a pseudo-Siamese network consisting of three steps:

- Extraction of feature matrices from each TF and target gene according to preprocessed datasets.
- Application of the GRU and DenseNet to obtain hidden time and spatial information.
- The regulatory and non-regulatory gene pairs are distinguished, and the prediction results are outputted.

With no prior knowledge of the regulators, we considered each TF as a potential network regulator for all genes.

PSGRN is a classification algorithm where the network inference problem is considered a binary classification task for each relationship, that is, whether TFs regulate target genes. Each classification task was performed by applying a pseudo-Siamese network, inferring GRNs from gene expression data by reverse reasoning. It simultaneously considers the time and spatial features of the regulatory relationship of expressed genes, extracts the time and spatial features, applies the deep learning framework, evaluates whether each TF has a regulatory relationship with target genes, and infers the entire GRN.

### Pseudo-siamese network

**Fig. 3:**
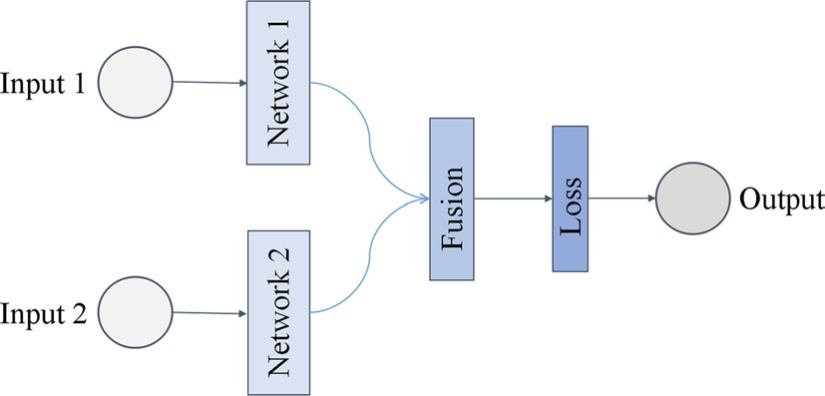
Pseudo-Siamese network structure [45]. In supervised learning, two or more input vectors represented by a network will maximize the distance of representation of different labels and minimize the distance of the same labels. A pseudo-Siamese network can evaluate the correlation between new vectors based on this strategy

The pseudo-Siamese network is a deep-learning framework with multiple inputs and a single output [45]. Two or more inputs were applied for feature extraction using specific neural network modules. The extracted features were then connected to the inner product and mapped to a new feature space. The loss function evaluates the correlation between the inputs. In supervised learning, a pseudo-Siamese network can be applied to maximize the distance between different labels and minimize the distance between the same labels to achieve accurate classification. Its structure is shown in Fig. 3.

A pseudo-Siamese network is suitable for dealing with situations in which correlations exist between different inputs. Pseudo-Siamese networks have been widely applied in many fields, such as natural language processing [46], image recognition [47], and signature analysis [48].

### Feature matrix extraction

PSGRN extract the feature matrix of the genes from each time-expression dataset. Denote $G= \left\{ G_{1}, G_{2}\dots ,G_{m} \right\}$, where *m* genes are in the gene expression profile, and $G_{i} = \left\{ G_{i,1}, G_{i,2}\dots ,G_{i,n} \right\}$, where the expression of $G_{i}$ at all *n* time points is represented. Data preprocessing of the original data before correlation analysis unifies the description and enhances comparability, ensuring that PSGRN can fully obtain the hidden state of the series. For each TF and target gene expression level, considering its time features, we applied the differential equation as the feature extraction method to obtain the difference matrix as follows:

1 $$\begin{aligned}&G_{i}^{'}=\left\{ G_{i,1}^{'},G_{i,2}^{'}, \dots ,G_{i,n-tl-1}^{'} \right\} \end{aligned}$$

2 $$\begin{aligned}&G_{i,j}^{'}=\left\{ G_{i,j+1}-G_{i,j},G_{i,j+2} -G_{i,j},\dots ,G_{i,j+tl}-G_{i,j} \right\} \end{aligned}$$

where *tl* is the time lag, and it calculates the series difference values for each time point to generate its feature matrix. The difference matrix captures gene expression changes after each time point. The feature dimension of $G_{i}$ is changed to $G_{i}^{'}=(n-tl-1)*tl$ after the series extraction.

The features of the original dataset include four different developmental stages, corresponding to double fertilization, endosperm, cell formation, and early differentiation stages. To analyze the mechanism of gene regulation more comprehensively, we set $tl=32$. Each gene feature was changed from 107 points to a $74*32$ matrix through the constructed feature matrix.

### Time feature learning

In this study, we conducted time-feature learning of the expression series, considering that transcription factors (TFs) and their target genes often exist at specific times during natural gene expression with little overall regulation. To achieve this, we employed the gated recurrent unit (GRU) as the unit of time feature learning.

The GRU is a variant of the vanilla RNN that incorporates a gating mechanism, similar to the long short-term memory (LSTM) unit. While both the GRU and LSTM have input and output gates similar to the vanilla RNN, the GRU removes the forgetting gate and applies the reset and update gates to retain valuable long-term memory and ignore unnecessary short-term noisy memories. Compared with LSTM, the GRU structure is more straightforward and requires less training, making it easier to implement. The standard architecture of the GRU network is available at Additional file 1. The internal GRU calculation formulae are as follows:

3 $$\begin{aligned} &r_{t} = \sigma \left( W_{r} \cdot \left[ h_{t-1},x_{t} \right] \right) \end{aligned}$$

4 $$\begin{aligned} &z_{t} =\sigma \left( W_{z} \cdot \left[ h_{t-1},x_{t} \right] \right) \end{aligned}$$

5 $$\begin{aligned} &h_{t}^{\prime }=\tanh \left( W_{h} \cdot \left[ r_{t}\odot h_{t-1}^{\prime },x_{t}\right] \right) \end{aligned}$$

6 $$\begin{aligned} &h_{t} = \left( 1-z_{t}\right) \odot h_{t-1}+z_{t}\odot h_{t}^{\prime } \end{aligned}$$

where $x_{t}$ is the current input vector of the GRU, and in PSGRN, it is the feature matrix of each gene expression level. $r_{t}$ and $z_{t}$ are the reset and update gates, respectively, and are computed using Eqs. 3 and 4. $\sigma$ and $\tanh$ denote sigmoid and the $\tanh$ activation functions, respectively. $h_{t-1}$, $h_{t}^{\prime }$ and $h_{t}$ are the previous, candidate, and current outputs, respectively. $w_{r}$, $w_{z}$, and $w_{h}$ are the weight matrices of the reset, update, and candidate gates, respectively, which are optimized during the training process.

We set the hidden dimension vector to 128. After the GRU studies the time features of the TF-target pairs, we concatenated them into one matrix and transferred them to the next module for spatial feature learning.

### Spatial feature learning

In this study, we utilized the DenseNet module to extract spatial features based on a feature matrix from a global perspective.

DenseNet is a popular CNN architecture [34]. As the successor to the ResNet architecture, DenseNet is composed of dense blocks and transition modules. DenseNet introduces a novel architecture that connects each layer to every other layer in a feed-forward fashion, enabling a maximum flow of information across all layers. The network is built using dense blocks, which contain multiple layers that are densely connected to each other. In each dense block, the feature maps from all preceding layers are concatenated to form the input to each layer, instead of being added or averaged as in other architectures. This enables each layer to have access to the collective knowledge of all preceding layers, which helps to alleviate the vanishing gradient problem and improves the feature reuse across layers. DenseNet also employs a transition layer between the dense blocks to reduce the spatial dimension of the feature maps, which improves computational efficiency and reduces overfitting. For the structure and submodules of DenseNet, please see Section II of Additional file 1 for more detail.

The utilization of deeper networks facilitates the extraction of high-dimensional features across regions, without being restricted by a series of correlation vectors. Additionally, the employment of more hierarchical connections helps to mitigate the problem of gradient vanishing and explosion, resulting in more effective feature propagation.

### Relatedness measurement

After the input features were extracted from these deep networks, the main features were extracted and outputted through the average pool and fully connected layers. In the output stage, PSGRN utilized binary cross-entropy (BCE) as the loss function to obtain binary results:

7 $$\begin{aligned} BCE=-\frac{1}{N} \sum _{i=1}^{N} y_{i}\log {\left( p\left( y_{i} \right) \right) +\left( 1-y_{i} \right) \log {\left( 1-p\left( y_{i}\right) \right) }} \end{aligned}$$

The BCE values were restricted to the range of $\left( 0,1 \right)$, where *N* represents the number of samples, $y_{i}$ represents the label of sample *i*, and $p_{i}$ represents the probability that sample *i* is predicted to be a positive label by the sigmoid function. PSGRN subsequently applied max-pooling and fully connected layers to obtain all features, followed by a sigmoid function as the classification measurement. The use of sigmoid instead of softmax was because softmax is only applicable to dichotomous issues, whereas in GRN classification, it is impossible to prove that two genes are completely unrelated based on current biotechnology. Therefore, this is not a straightforward dichotomous issue. The logical value of $\left( 0,1 \right)$ in GRN classification inference represents the relationship between “currently not verified correlation” and “currently verified correlation,” which are not absolute. Thus, PSGRN applied the sigmoid function to transform the output to the range of $\left( 0,1 \right)$ and then employed various thresholds for classification based on different techniques.

## Results

### Model training and datasets partition

According to GRNs from the real world, we marked the gene pairs of TFs and target genes as positive sample 1, and those without regulatory relationships were marked as negative sample 0. A GRN is a sparse network in which the number of positive labels is much less than that of negative labels, which belongs to the problem of unbalanced samples. This problem is reflected in the network density of previous datasets. To comprehensively analyze the performance of our model, we divided the relationship of each gene pair in each dataset into training, test, and validation sets at a ratio of 3:1:1 and ensured that the proportion of positive and negative samples after the division was consistent with the original data set. PSGRN were implemented based on the TensorFlow platform, and with other compared models, all these experiments were run on a computer configured with Intel Xeon 16896k + 64GB RAM + 4 * NVIDIA GTX 1080ti.

**Fig. 4:**
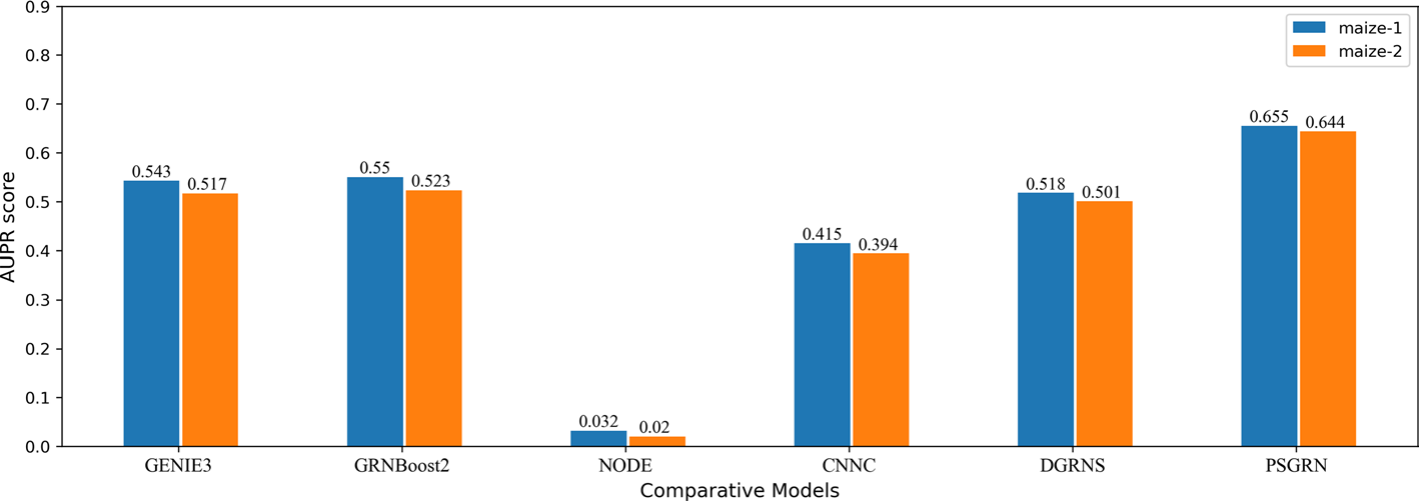
Performance comparison of different models. The color corresponds to each dataset, and the groups represent different models. The AUPR scores obtained by each model are marked above each designated column

### Evaluating metrics

Most existing methods for inferring gene regulatory networks rely on pre-training their models and obtaining scores to predict the relationship between TFs and their target genes. The score reflects the confidence level of the regulatory relationship, and the network is usually determined by setting a threshold.

In this study, we analyzed the expression dataset of maize seeds and constructed the time series of TFs and their target genes as inputs. We obtained the output coefficients $\alpha$ in the range of $\left( 0,1 \right)$. For the two-classification problem, we conducted a comprehensive analysis of all output coefficients and determined an appropriate threshold $\theta$. We classified the output with $\alpha \ge \theta$ as positive and $\alpha < \theta$ as negative.

In most previous studies, the area under the receiver operating characteristic (ROC) curve (AUROC) and the area under the precision-recall (PR) curve (AUPR) scores were used as evaluation criteria for GRN inference models. The PR curve was drawn using precision and recall rates, and the ROC curve was drawn using false positive rate (FPR) and true positive rate (TPR) rates. However, we found that AUROC can only evaluate balanced problems, whereas GRN inference is highly unbalanced, with far fewer interacting gene pairs than non-interacting pairs. Therefore, we used AUPR as the metric to evaluate the highly unbalanced datasets of the results of each model.

Our model’s experimental results provide all prediction edges and their corresponding weights. The weight indicates the credibility of the regulatory relationship at the edge. We evaluated the performance of each model by calculating the corresponding accuracy and recall rates by specifying an appropriate threshold. Finally, we obtained the corresponding relationship through comprehensive analysis.

### Performance evaluation

Among the many unsupervised models, we selected GENIE3 [27], GRNBoost2 [28], and nonlinear ordinary differential equations (NODE) [26]. These are all typical methods with a significant impact on inferring GRNs from gene expression series with unsupervised learning. Similarly, for supervised learning, we compared the PSGRN with CNNC [31] and DGRNS [32], which are related to our method and significantly outperform other methods, such as PIDC [49], SCODE [50], and ARACNe [51].

**Table 2:** Running time comparison of different models

Model	GENIE3	GRNBoost2	NODE	CNNC	DGRNS	PSGRN
Maize-1	198.07 s	34.65 s	37.12 s	22.13 min	210.47 min	28.70 min
Maize-2	355.23 s	62.34 s	72.54 s	38.75 min	480.19 min	73.49 min

The performance comparison results of various methods based on their AUPR values on the two-scale datasets are presented in Fig. 4, and their running time is shown in Table 2. PSGRN achieved AUPR values of 0.655 and 0.664, respectively, from the analysis of the two datasets, which significantly exceeded those of the other models. Our model considers both time and spatial features, rather than treating expression data as a whole. On the other hand, NODE is specifically designed for time-expression data and runs the fastest among all models. However, it yielded the poorest performance because it views gene interactions as a whole process, whereas such interactions occur at specific moments, indicating that NODE is not appropriate for GRN inference in natural organisms.

**Fig. 5:**
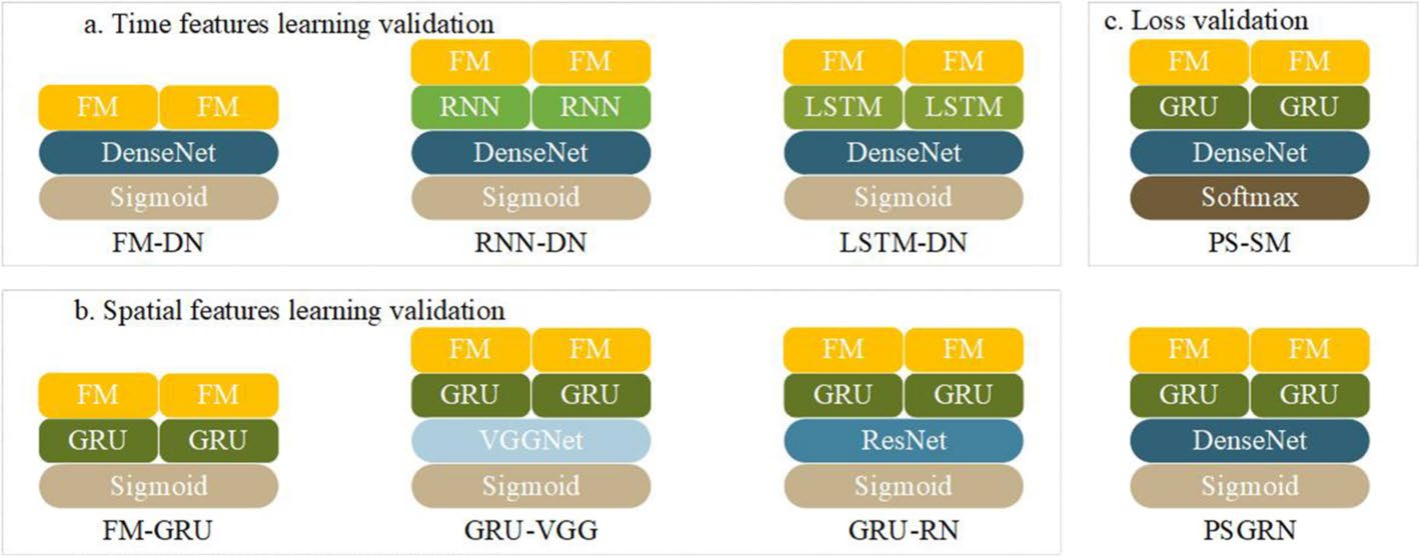
Module effectiveness validation of PSGRN. This study aimed to verify the optimal performance of each module of PSGRN by comparing different modules. In order to evaluate the time feature learning module, we removed the GRU module and implemented either RNN or LSTM to replace it. Similarly, the spatial feature learning module was also substituted by either VGGNet or ResNet in order to assess its performance. Additionally, for part c, the loss function was replaced with the softmax function instead of the sigmoid function. These modifications were made to better understand the effectiveness of each module within PSGRN

The performances of GENIE3 and GRNBoost2 were not ideal as they are trained according to random subsets and do not consider all genes. As the successor of GENIE3, GRNBoost2 applied gradient-boosted decision trees and a regularized early stopping strategy to prevent the model from overfitting, making it insensitive to dropout events. GRNBoost2 does not solve the sparse and unstable problems of GENIE3.

CNNC and DGRNS have achieved impressive results on unbalanced datasets, which can be attributed to the successful integration of information theory and deep learning. Specifically, DGRNS has introduced PCC on top of the CNNC model, leading to superior performance compared to the original CNNC model. However, limitations exist due to the short time intervals between data points in the datasets, and the Pearson coefficient’s inability to capture non-linear relationships between TFs and target genes. Furthermore, the two-layer CNN structure used in CNNC and DGRNS may not fully capture the underlying information. Additionally, the computation of PCC in DGRNS is time-consuming (nearly 90%). Despite the extended running time, the marginal improvement achieved by DGRNS over other models is not significant, rendering the cost unjustifiable.

In summary, PSGRN runs slower than other models except for DGRNS but achieves the best results. The comparison of PSGRN with other models indicates that the performance of PSGRN in inferring GRNs is highly effective. The results highlight the importance of time and spatial feature learning in GRN inference, and the significance of considering time series in sections in GRN inference. These findings demonstrate that PSGRN can significantly improve the accuracy and efficiency of GRN inference and provide insights into the development of future deep-learning models for GRN prediction.

### Model validation

#### Robustness validation

To validate the robustness of PSGRN, this study employs two verification strategies, namely, introducing noise to the experimental dataset and extracting subsets from the experimental dataset. The study investigates the difference between the PSGRN model and the original experimental results in the presence of noise and incomplete data sets. The validation experiment is based on the maize-1 dataset.

**Fig. 6:**
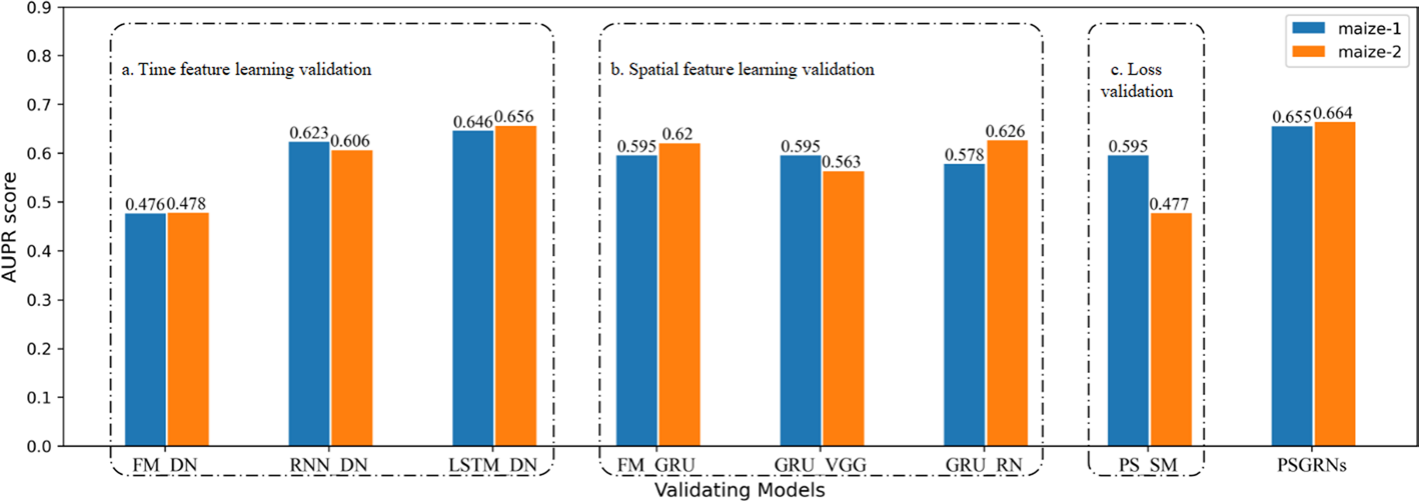
Validation of each module. To validate the effectiveness of our PSGRN model, we conducted ablation experiments on three critical components: the time feature learning module, spatial feature learning module, and classification module. By removing each module and evaluating the performance of the modified model, we assessed the contribution of each module to the overall performance of PSGRN. This analysis allowed us to demonstrate the superiority of our model and provide insights into the specific components that contribute to its success

To introduce data noise, this study randomly selects a TF-target relationship pair in the data acquisition stage, duplicates the time expression sequence of the two, and randomly selects the time expression sequence of a gene in the maize-1 dataset and the original time sequence of TF and Target, respectively, to generate noise samples. we selects 5% of the TF-target pairs in the maize-1 dataset randomly to construct noise samples, forming a noise dataset. we carries out ten repeated experiments, records the AUPR value of ten experiments, takes the mean value, and records the variance of ten experimental results for analysis.

For the analysis of incomplete datasets, this study adopts the resampling analysis method for verification. It randomly selects 70% of the data from the maize-1 dataset to generate an incomplete dataset. Then, it inputs the obtained sub-dataset into PSGRN for evaluation. we also conducts ten repeated experiments, records the AUPR values of all experiments, takes the mean value, compares it with the performance of PSGRN in the original dataset, and records the variance of the resampling experiment results for analysis.

**Table 3:** The result of PSGRN in the noisy and incomplete dataset

Method	AUPR (mean)	AUPR (variance)
Noise	0.633	$1.249\times 10^{-4}$
Incomplete	0.631	$1.720\times 10^{-4}$
Original	0.655	$1.492\times 10^{-4}$

Table 3 illustrates the comparison between the two validation methods and the results of PSGRN in the maize-1 dataset. It can be observed that PSGRN still achieves similar results to the original dataset in the presence of noise and incomplete data. This is attributed to the PSGRN model’s sufficient depth of layers and strong learning ability, allowing it to accurately identify gene interactions even in the presence of noise and incomplete data.

After verifying the robustness of the model, we will further verify the effectiveness of each module in the model and analyze the effectiveness of each layer in the model. These verifications will help confirm the PSGRN model’s performance and provide more reliable support for its practical applications.

#### Effectiveness validation

Many deep learning models for time and spatial feature learning with good performances have been proposed. To verify the optimal performance of the PSGRN, we applied various widely used methods to build different models and compared their performance in time and spatial feature learning. The structure of each model is illustrated in Fig. 5.

To verify the effectiveness of time-feature learning, we modified the PSGRN and built three models (Fig. 5a):

- Removal of the temporal feature learning module so that the feature matrix of the TFs and target genes are directly inputted into DenseNet, called FM-DN. Here, FM represents the feature matrix, and DN represents the DenseNet module.
- Application of vanilla RNN as the time feature learning module, called RNN-DN.
- Application of LSTM as a time feature learning module, called LSTM-DN.

Similarly, we constructed the following models for the validity test of the spatial features (Fig. 5b):

- Construction of the FM-GRU model, which removes the spatial feature learning module and directly calculates the correlation of the GRU outputs.
- Replacement of DenseNet with VGGNet [52] and ResNet, which are called GRU-VGG and GRU-RN, respectively, where RN is the ResNet module.

It is worth mentioning that in the FM-GRU model, the GRU output and target genes in TFs are two matrices. Considering that there is no spatial feature learning part to consider these matrices comprehensively, we changed the previous matrix series to multiplication. In addition, to verify the effectiveness of the sigmoid function in model classification, we replaced the last sigmoid function in the PSGRN with the softmax function, called PS-SM (Fig. 5c). Here, SM represents the softmax function.

We evaluated their performance on maize-1 and maize-2 datasets. The results are shown in Fig. 6.

Among all the compared models, PSGRN achieved the best results. Notably, the performance of PSGRN was significantly improved after replacing the softmax function with the sigmoid function, indicating that the softmax function is unsuitable for GRN inference. The softmax function maps the output to the range of $\left( -\infty ,+\infty \right)$ and then normalizes it, increasing the distance between different labels as much as possible. However, this strategy is often unsuitable for GRN inference, as researchers cannot evaluate whether there is “no interaction” between two genes due to limitations in current technology. As stated in Section “Relatedness Measurement”, label 0 indicates “no correlation found at present”, not “absolutely irrelevant”. Over-maximizing the sample distance between label 1 and label 0 is not conducive to discovering unknown interactions. We expected to obtain the probability value of the interaction between two genes, representing the reliability of the corresponding category. The sigmoid function maps the actual output number field to the practical real number space in the range of $\left( 0,1\right)$ and represents a probability distribution that is more suitable for GRN inference.

**Fig. 7:**
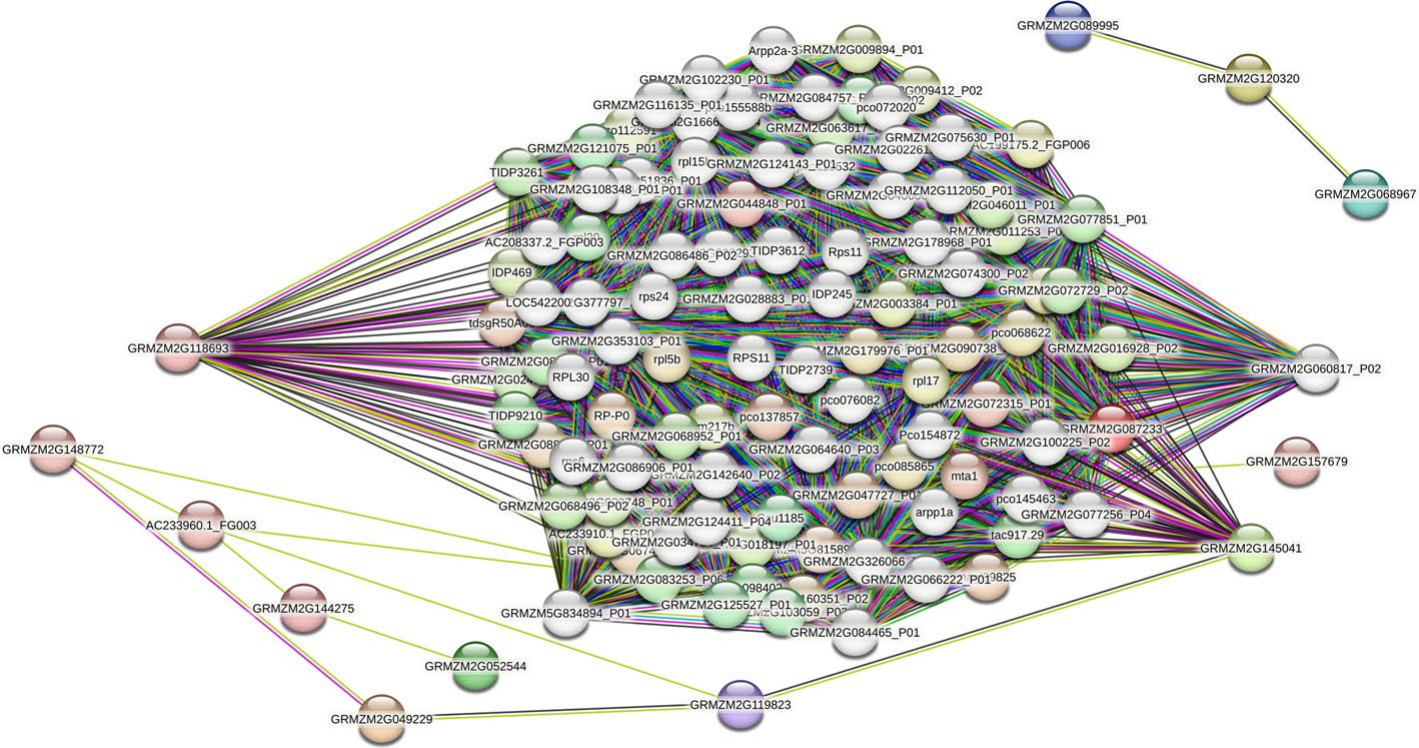
Illustrate of interactions of top 15 TFs. To validate the accuracy of our model, we employed the STRING database to perform a search on the top 15 TFs and generate a PPI network diagram. The nodes in the network represent maize genes, while the colored lines depict the sources of the interactions, such as experiments, text mining, databases, and co-expression analysis. Our analysis highlighted GRMZM2G145041 and GRMZM2G118693, which exhibited a high number of edges in the PPI network, suggesting their potential involvement in the maize gene regulatory network. These results provide further evidence for the accuracy and applicability of our model in predicting gene interactions in maize

In the field of time feature learning, FM-DN achieves the worst performance, and RNN-DN and LSTM-DN have significantly improved compared with FM-DN, which fully illustrates the necessity of time feature learning. Compared with GRU, the vanilla RNN lacks the time memory module, which is also the reason for the relatively poor performance of RNN-DN. Compared with the GRU, LSTM has one more gate. Hence, LSTM performs better than GRU for long-time series learning. However, the gene expression series is not a long-time series, and too many memory modules will increase the redundant information. The GRU can reduce redundant information and obtain a better performance than LSTM.

The performance of each validation model differed in the field of spatial feature learning. Without spatial feature learning, FM-GRU outperformed GRU-RN in the maize-1 dataset and GRU-VGG in the maize-2 dataset but was still inferior to PSGRN as it is difficult to capture features in a short-term gene expression series. When the dataset is relatively small, a shallower network can reduce the overfitting phenomenon and retain more features. Larger datasets must apply deeper networks to capture inherent features. VGGNet and ResNet can perform only one of the feature transmissions and deep networks required for feature extraction. DenseNet can solve this problem; as more feature connections are set, the output of each layer can be inputted into a deeper network, which also effectively alleviates the problems of gradient vanishing and exploding. This is why the PSGRN achieved better results than the other models.

### Network analysis

In this study, we utilized PSGRN to infer the gene regulatory network (GRN) of maize-1. The trained model was applied to predict interactions in the maize-1 dataset, and we set a threshold value $\mu$ as the absolute value of the 10000th most significant coefficient in result *A* to account for the density of the inferred GRN. Based on this threshold, we calculated the number of interactions ($A\ge \mu$) and non-interactions ($A<\mu$), resulting in 6914 true positives (TP), 38108 true negatives (TN), 3308 false positives (FP), and 2776 false negatives (FN).

Taking inspiration from the analysis conducted by Matsumoto et al. [50], this study focuses on the analysis of positive edges of each TF under the PSGRN prediction, with a specific focus on the top 15 TFs having the largest number of edges. The complete results and analysis notes are available at https://github.com/cirila9/PSGRN. These TFs are highly expressed during double fertilization and coenocyte formation in maize seeds, respectively. The current investigation has identified these TFs as being related to other genes in the GRN. While these genes are not considered to be indispensable for the maintenance of pluripotency in the nucellus and endosperm cells of maize [53], previous studies have demonstrated their significance in the process of differentiation. For instance, these TFs are known to restrict the lineage-specific functions of other genes during differentiation through histone methylation [54]. Moreover, GRMZM2G145041 is presumed to play a potential role in DNA methylation, while GRMZM2G119823 is involved in regulating genomic imprinting [55]. Hence, it is reasonable to conclude that GRMZM2G119823 and GRMZM2G145041 have an impact on the expression of genes in maize endosperm.

To gain deeper insights into our findings, we constructed a protein-protein interaction (PPI) network using the Search Tool for the Retrieval of Interacting Genes/Proteins (STRING) database [56]. We focused on the top 15 TFs in the maize-1 dataset and queried the STRING database to generate a functional PPI network between the TFs and other proteins, following transcription and expression. The nodes in the network represent maize genes, and the colored lines depict the sources of the interactions, such as experiments, text mining, databases, and co-expression analysis. The partial results of our analysis are presented in Fig. 7.

Our investigation revealed that PSGRN could effectively identify genes in the network, include GRMZM2G118693 and GRMZM2G145041, suggesting their definite involvement in the maize gene regulatory network. These findings provide further evidence for the accuracy and applicability of our model in predicting gene interactions in maize. Additionally, we identified novel potential regulatory relationships, and the inferred network interactions were consistent with those present in the standard network. Notably, PSGRN predictions not included in the database are not necessarily incorrect, as many gene regulatory relationships remain unverified by biological experimentation. PSGRN can identify numerous potential rules between genes based on existing information and can save researchers significant time in identifying the next direction for exploration. Our study provides a foundation for supplementing biological data and identifying critical regulators of differentiation in maize.

## Discussion and conclusion

This study proposes a deep learning framework called a PSGRN for GRN inference. It is based on supervised learning and a pseudo-Siamese network. A GRU and DenseNet were applied for processing time-series data and analyzing feature matrices, respectively, effectively improving the model’s performance.

To comprehensively assess the performance, we compared PSGRN with existing methods, including both representative supervised and unsupervised learning methods. Our model achieved the best performance among the existing advanced models on two real maize datasets. The experiments showed advantages in processing the expression profile data and identifying potential regulatory relationships.

The inference in this study infers the edges for a given dataset with TF and target tags. In PSGRN, unknown relationships can be inferred by mining the correlations between known regulatory relationships. Thus, exploring the temporal and spatial features of the dataset through deep learning helps us mine the internal relationships among them, providing insight into the functional understanding of GRNs.

The validity of the GRU and DenseNet modules was verified, and the comparative results revealed the necessity of setting these two modules and their excellent performance in terms of time and space learning. In addition, we experimentally verified that the sigmoid function is more suitable for GRN inference than the softmax function.

All aspects of biological subsystems, such as scale-free properties, structural properties, motifs, and environmental factors, may affect cell interactions. PSGRN still lack prior knowledge, such as specifying the maximum possible number of candidate relationships for each gene and determining which genes play a regulatory role in a specific network. Inducing more domain-specific knowledge of PSGRN may help predict the network more accurately and make PSGRN a useful model for future work.

## Supplementary information

**Additional file 1.** Supplementary Material.

## Data Availability

The supplementary data can be accessed online to NCBI database (https://www.ncbi.nlm.nih.gov/). The gene expression dataset of maize seed is saved in PRJNA505095 and the GRN dataset of maize leaf is saved in PRJNA518749. This study’s data, code, and results are available at https://github.com/cirila9/PSGRN.
