# Supplementary material for: A gene regulatory network inference model based on pseudo-siamese network
Source: BMC Bioinformatics. 2023 Apr 21;24:163. doi: 10.1186/s12859-023-05253-9 (PMC10122305; doi:10.1186/s12859-023-05253-9)
Supplement: Supplementary file 1 — Additional file 1. Supplementary Material. [file 12859_2023_5253_MOESM1_ESM.pdf]

Additional File of "A model of Gene Regulatory Networks inference based on Pseudo-Siamese Network"

I. Time Feature Learning

We used the Gated Neural Unit (GRU) [1] as the unit of time feature learning.

As a variant of the vanilla RNN, the long short-term memory (LSTM) unit adds a gating mechanism and benefits from it [2]. The GRU's input and output gates are similar to those of the vanilla RNN, and its intrinsic idea is similar to that of the LSTM. Compared with LSTM, the GRU removes the forgetting gate, applies the reset and updates gates to retain valuable long-term memory, and ignores unnecessary short-term noisy memories. Meanwhile, the GRU structure is more straightforward and requires less training than LSTM; therefore, it is easier to implement. The GRU structure is illustrated in Fig. 1.

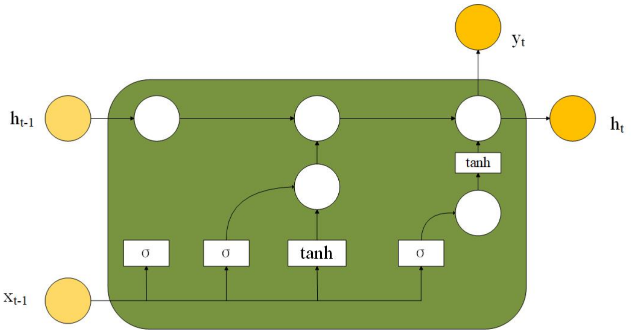

Fig. 1. The structure of GRU containing reset and update gates [1]. First, the GRU obtains the two gating states through the last transmitted state h_{t-1} and the input x_t of the current node. After obtaining the gating signal, it resets the gating to obtain $h^{'}_{t-1} = h_{t-1}$, connects the $h^{'}_{t-1}$ with x_t, and then reduces the data to the range of $(-1, 1)$ through the activation function $\tanh$ to obtain h_t.

Typically, there is little overall regulation between the TFs and their target genes during natural gene expression; they often exist only at specific times. Therefore, we conducted time-feature learning of the expression series. After the N-dimensional (ND) feature matrix input, the GRU extracts its features. The internal GRU calculation formula is as follows:

$$r_t = \sigma\left(W_r \cdot [h_{t-1}, x_t]\right) \tag{1}$$

$$z_t = \sigma\left(W_z \cdot [h_{t-1}, x_t]\right) \tag{2}$$

$$h'_t = \tanh\left(W_h \cdot \left[r_t \odot h'_{t-1}, x_t\right]\right) \tag{3}$$

$$h_t = (1 - z_t) \odot h_{t-1} + z_t \odot h'_t \tag{4}$$

where x_t is the current input vector of the GRU, and in PSGRN, it is the feature matrix of each gene expression level. r_t and z_t are the reset and update gates, respectively, and are computed using Eq. 1 and Eq. 2. σ and $\tanh$ denote sigmoid and the $\tanh$ activation functions, respectively. h_{t-1}, h'_t and h_t are the previous, candidate, and current outputs, respectively. w_r, w_z, and w_h are the weight matrices of the reset, update, and candidate gates, respectively, which are optimized during the training process.

We set the hidden dimension vector to 128. After the GRU studies the time features of the TF-target pairs, we concatenated them into one matrix and transferred them to the next module for spatial feature learning.

II. Spatial Feature Learning

In this study, the DenseNet module [3] was applied to extract spatial features based on a feature matrix from a global perspective.

A vanilla CNN comprises convolution, pooling, and fully connected layers, and the output is transformed into a probability distribution through the loss function. An important role of the convolution kernel is to perform region and channel crossing of the input information. With the deepening of the network, the information obtained becomes more abstract, and the advanced features are easier to detect. However, deep networks cause gradient vanishing and exploding. The residual neural network (ResNet) [4] solves these problems by introducing residual learning, making a deeper network possible.

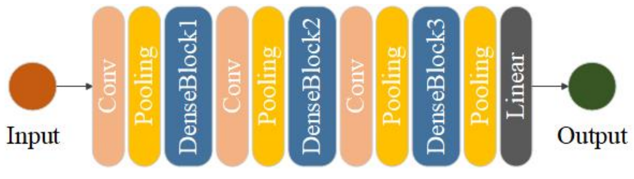

Fig. 2. The structure of DenseNet [3]. It establishes dense connections between all the front layers and the back layers. In addition, DenseNet realizes feature reuse by connecting features on the channel, which enables DenseNet to perform better than ResNet with fewer parameters and computational costs.

The DenseNet structure is illustrated in Fig. 2. As the successor of ResNet, DenseNet is mainly composed of DenseBlock and transition modules. Its structure is similar to that of ResNet and inherits the advantage of being good at capturing

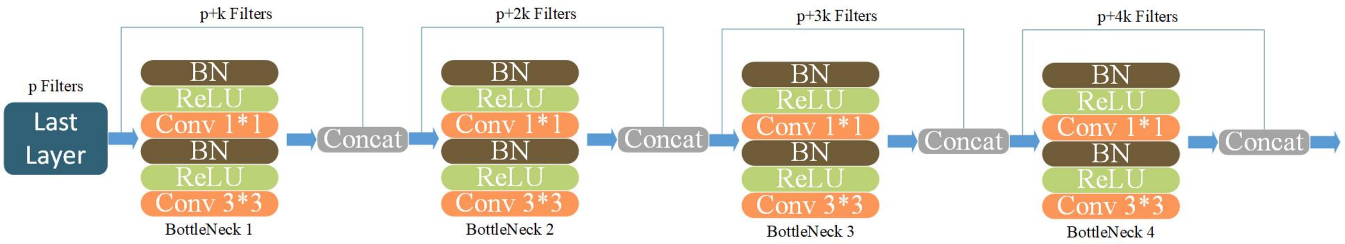

Fig. 3. The internal structure of DenseBlock [3]. It applies the order of activation function in the front and the convolution layer in the back. The dense connection is adopted between layers. By advancing the hyperparameter K, the module can get K feature graphs after the convolution of each layer in all DenseBlocks. Assuming that the channel number of the feature graph of the input layer is p, the channel number of the input layer q is $p+k\cdot(q-1)$. As the number of layers increases, the features graph size of each layer remains the same.

valuable features. Simultaneously, it applies a deeper convolution network and connects the output of each layer with the input of each previous stage.

The vanilla CNN must go through a pooling layer to reduce the size of the feature map. To solve this problem, the DenseBlock structure and the transition are applied in DenseNet. First, DenseNet performs large-scale convolution and pooling layers. It then connects several successive submodules (DenseBlock, bottleneck layer, and transition layer), in which the transition layer includes one $1*1$ convolution kernel and one $2*2$ pooling kernel. It connects two adjacent DenseBlocks and reduces the size of the feature map.

Denseblock is a module that contains many layers. Each layer's feature diagram size was the same, and a dense connection mode was adopted between the layers. Each DenseBlock has the same combination and is applied as input to the next layer, as shown in Fig. 3.

One ReLU activation layer and one batch normalization (BN) layer [5] were applied between each $1*1$ and $3*3$ convolution kernel. These layers were set to normalize the data to a unified interval, reducing the data divergence and the learning difficulty of the network.

The last layers are the pooling layer and the fully connected layer. Compared with ResNet, DenseNet proposes a more radical dense connection mechanism by connecting all layers. Specifically, each layer receives all the preceding layers as its additional input:

$$F_l = H_l\left([f_1, f_2, \ldots, f_{l-1}]\right) \tag{5}$$

where F_l represents the non-linear transformation function, and $f_1, f_2, \ldots, f_{l-1}$ represents the features extracted in the previous multi-networks structure.

These deeper networks are conducive to extracting high-dimensional features across regions without being limited by a series of correlation vectors. Applying more hierarchical connections to slow down gradient lifting and explosions has a stronger feature transitivity.

III. Evaluating Metrics

Most methods predict the relationship between a series of TFs and their target genes after pretraining their model and obtaining the corresponding scores to evaluate performance. The higher the score, the higher the confidence that there is a regulatory relationship between them. A network is usually determined by specifying a threshold.

By analyzing the expression dataset of maize seeds, this study constructed the time series of the TFs and their target genes as inputs and obtained the coefficients of output α in the range of $(0,1)$. For the two-classification problem, we analyzed all the output coefficients comprehensively, specified an appropriate threshold θ, and classified $\alpha \geq \theta$ as the positive label and $\alpha < \theta$ as the negative label.

Different requirements result in different thresholds. Based on the model prediction, we obtained four possible results:

- True-positive (TP): The edge exists in the gold standard and is predicted by the model.
- True-negative (TN): The edge does not exist in the gold standard and is not predicted by the model.
- False-positive (FP): The edge does not exist in the gold standard but is predicted to exist by the model.
- False-negative (FN): An edge exists in the gold standard, but the model predicts it does not exist.

Therefore, the precision and recall rates according to these conditions can be calculated as follows:

$$TPR = \frac{TP}{TP+FN} \tag{6}$$

$$FPR = \frac{FP}{FP+TN} \tag{7}$$

$$Recall = \frac{TP}{TP+FN} \tag{8}$$

$$Precision = \frac{TP}{TP+FP} \tag{9}$$

The TPR is the proportion of the number of true-positive samples detected to the total number of true-positive samples. The FPR is the proportion of false-positive samples detected to the number of true-negative samples. The recall rate is the proportion of correctly inferred samples that are accurate for the overall positive samples. The precision rate refers to the proportion of correctly inferred samples to all samples inferred as positive.

Most previous studies applied the area under the receiver operating characteristic (ROC) curve (AUROC) and the area

under the precision-recall (PR) curve (AUPR) scores as the evaluation criteria of the GRN inference model. The precision-recall (PR) curve was drawn using the precision and recall rates. An ROC curve was drawn using the FPR and TPR rates. We found that the AUROC can only evaluate balanced problems. However, GRN inference is a highly unbalanced problem. The number of interacting gene pairs in the GRN inference was far less than that without interaction. Therefore, we used the AUPR as the metric to evaluate the highly unbalanced datasets of the results of each model.

The experimental results of our model provide all the prediction edges and their corresponding weights. The credibility of the regulatory relationship at the edge increases with weight. The performance of each model can be accurately evaluated by calculating the corresponding accuracy and recall rates by specifying an appropriate threshold. Finally, we obtained the corresponding relationship through a comprehensive analysis.

References

[1] K. Cho, B. Van Merriënboer, D. Bahdanau, and Y. Bengio, "On the properties of neural machine translation: Encoder-decoder approaches," *arXiv preprint arXiv:1409.1259*, 2014.

[2] S. Hochreiter and J. Schmidhuber, "Long short-term memory," *Neural computation*, vol. 9, no. 8, pp. 1735–1780, 1997.

[3] G. Huang, Z. Liu, G. Pleiss, L. Van Der Maaten, and K. Weinberger, "Convolutional networks with dense connectivity," *IEEE transactions on pattern analysis and machine intelligence*, 2019.

[4] K. He, X. Zhang, S. Ren, and J. Sun, "Deep residual learning for image recognition," in *Proceedings of the IEEE conference on computer vision and pattern recognition*, 2016, pp. 770–778.

[5] S. Ioffe and C. Szegedy, "Batch normalization: Accelerating deep network training by reducing internal covariate shift," in *International conference on machine learning*. PMLR, 2015, pp. 448–456.
